# Associations of particulate matter and its components with emergency room visits for cardiovascular and respiratory diseases

**DOI:** 10.1371/journal.pone.0183224

**Published:** 2017-08-15

**Authors:** Sung-Hee Hwang, Jae Young Lee, Seung-Muk Yi, Ho Kim

**Affiliations:** 1 Graduate School of Public Health, Seoul National University, Seoul, South Korea; 2 Institute of Health and Environment, Seoul National University, Seoul, South Korea; The Ohio State University, UNITED STATES

## Abstract

Numerous studies have revealed an association between particulate matter (PM) and emergency room (ER) visits, although few studies have investigated the association between health and PM components. The present study evaluated the associations of ER visits for cardiovascular and respiratory diseases with PM_2.5_ components, including organic carbon (OC), elemental carbon (EC), and ion species (SO_4_^2-^, NO_3_^-^, and NH_4_^+^). Statistical analyses were performed using the time-series approach, and generalized linear models with natural spline functions were used to adjust for the non-linear relationship between the confounders and ER visits. Our single-pollutant models revealed that the greatest increase in cardiovascular ER visits was associated with NH_4_^+^ (relative risk: 1.05; 95% confidence interval: 1.01–1.09), which was followed by OC, SO_4_^2-^, NO_3_^-^, and EC. The associations of cardiovascular ER visits with EC and OC varied according to age and sex, with elderly and female patients exhibiting stronger associations. Lagged SO_4_^2-^ was associated with respiratory ER visits. To the best of our knowledge, this is the first study to evaluate the associations between ER visits and PM components in South Korea. As PM components are related to traffic and industrial sources, and exhibited positive associations with ER visits, our results may help improve air pollution regulation and public health.

## 1. Introduction

Concerns regarding the health effects of air pollutants have been increasing, and several studies have revealed that adverse health effects are related to increasing concentrations of particulate matter (PM) [1–8]. Previous studies have also examined the association between mortality and PM concentrations, and typically showed that mortality increases with greater population-level exposure to air pollutants [9–16]. Furthermore, researchers have demonstrated that emergency room (ER) visits for cardiovascular and respiratory diseases may increase with elevated PM concentrations [17–25]. Moreover, South Korean studies have revealed an association between PM concentrations and ER visits for cardiovascular and respiratory diseases [26, 27].

Although there is increasing evidence regarding the effects of PM, there is uncertainty regarding the association of health with exposure to specific PM components. In addition, only a few studies have evaluated whether PM components influence the number of ER visits for specific diseases. Chen et al. [28] found that PM_2.5_ components were more closely related to ER visits for hemorrhagic stroke, compared to the mass of the PM_2.5_. Furthermore, Qiao et al. [29] have reported a positive association between ER visits and PM_2.5_ components, such as organic carbon (OC) and elemental carbon (EC) from fossil fuel combustion. However, despite the influence of PM components on ER visits and health, studies regarding this topic are limited by the difficulty of collecting and analyzing PM component data. Therefore, this study evaluated the associations of PM components with ER visits for cardiovascular and respiratory diseases in Seoul, South Korea (2010–2013). To the best of our knowledge, this is the first study to evaluate this association in South Korea using time-series analysis.

## 2. Materials and methods

### 2.1 Data collection

The present study was approved by the Seoul National University Institutional Review Board (IRB No: 1501/001-013). This study evaluated daily data (April 17, 2010 to May 10, 2013) regarding PM and ER visits for cardiovascular diseases (ICD-10: I00-I99, G45, G46, M30, M31, R58) and respiratory diseases (ICD-10: J00-J99, I46, I47, I48, I49) in Seoul, South Korea. The ER visit data were obtained from the National Emergency Department Information System (NEDIS), and the hourly PM_10_ and PM_2.5_ (PM with diameters of ≤10 μm and ≤2.5 μm, respectively) concentration data were obtained from the National Institute of Environmental Research. The Korea Meteorology Administration provided daily meteorological data, including temperature (°C) and relative humidity (%).

NEDIS is a computerized system that is designed to transmit and analyze emergency care information from 332 emergency medical institutions in 16 metropolitan cities in Korea (based on 2013 values). Fifty-four of the emergency medical institutions were in Seoul. To extract emergency medical center data, we analyzed data from 30 hospitals in Seoul. In addition, NEDIS provides data at the individual level, such as age, sex, primary diagnostic code, ER visit date(s), reasons for the ER visit, and hospital district. Anonymized ER visit data were classified according to the discharge diagnosis.

For our analyses, we considered the average daily concentrations of PM_10_ and PM_2.5_. The PM components were defined as carbon species (EC and OC) and ion species (NO_3_^-^, NH_4_^+^, and SO_4_^2-^), and related data were obtained during 24-h periods using ambient air samples that were collected on the rooftop of the former School of Public Health building (37.5°N and 127.00°E), which is located in the center of Seoul, between April 2010 and March 2013. The distance between the farthest hospital and the measurement site was 14.09 km. The concentrations of carbon species and ion species were analyzed using thermal and optical transmittance (Sunset Laboratories, Tigard, OR) and ionic chromatography (Dionex DX-120; Thermo Fisher Scientific, Inc., Cambridge, UK), respectively. More detailed information regarding the measurement procedures have been reported by Heo et al. [30] and Kim et al. [31].

### 2.2 Statistical analysis

To evaluate the relationships between ER visits and concentrations of PM components, we used a generalized linear model based on the assumption of a quasi-Poisson distribution. The model included various controlling factors, such as influenza status, day of the week (DOW), holiday, and meteorological variables (temperature and relative humidity):

$$log(\mu_{t})=\alpha +{Component}_{t}+factor({DOW}_{t})+factor({Influenza}_{t})+factor({Holiday}_{t})+ns({Temp}_{1-t},df=6)+ns({RH}_{t},df=3)+ns(Time,df=7/year)$$ (1)

In this model, μ_*t*_ is the expected number of ER visits on day t, Component_*t*_ is the concentrations of the PMs and their components on day t (μg/m^3^), and α is the intercept of the model. DOW_*t*_ is the day of the week, which was evaluated as a categorical variable on day t (Monday, Tuesday, Wednesday, Thursday, Friday, Saturday, and Sunday). Influenza_*t*_ was evaluated as a categorical variable on day t (presence: 1, absence: 0), Holiday_*t*_ was evaluated as a categorical variable on day t (holiday: 1, non-holiday: 0), and *ns*(Temp_1−*t*_,*df* = 6) was the natural cubic spline function of time with 6 degrees of freedom (df) per year. To control the delayed association between temperature and ER visits, we applied a 5-day moving average for daily temperature. *ns*(RH_*t*_,*df* = 3) was the natural cubic spline function of humidity with 3 df per year. We used a df of 7 per year for the natural cubic spline function to control for long-term trend and seasonality. Akaike’s information criterion for over-dispersion was applied to examine the statistical model [32]. The choice of the model, the spine functions, and the adjustment for time and seasonality were introduced by Peng et al. [21], Heo et al. [33] and Qiu et al. [34]. PM_10_, PM_2.5_, and their components were evaluated by using single-pollutant models. In addition, we created multi-pollutant models that contained each pollutant while simultaneously controlling for the other components. A sensitivity analysis was performed to determine the stability of the fitting model. All statistical analyses were performed using R software.

## 3. Results

Table 1 describes the basic characteristics of the ER visits for cardiovascular and respiratory diseases between April 17, 2010 and May 10, 2013. We identified 149,452 ER visits for cardiovascular diseases (133.44 visits/day) and 453,868 ER visits for respiratory diseases (405.24 visits/day). Compared to female patients, male patients had a higher average value for cardiovascular and respiratory diseases. Elderly patients (≥65 years old) had lower mean values for cardiovascular and respiratory diseases, compared to younger patients (<65 years old). However, the ER visits for both cardiovascular and respiratory diseases distinctly increased over time among elderly patients (≥65 years old). Compared to 2010, the cardiovascular ER visits among elderly patients increased by 15.51% in 2013. The increases were 9.73% for female patients, 8.72% for male patients, and 3.23% for younger patients. The respiratory ER visits among elderly patients increased by 66.85% in 2013, compared to 2010. The increases were –7.62% for female patients, –12.75% for male patients, and –16.04% for younger patients. Figs A and B in S1 File show the time series distributions of cardiovascular and respiratory ER visits according to sex and age.

**Table 1:** Characteristics of emergency room visits for cardiovascular and respiratory diseases between April 17, 2010 and May 10, 2013.

Variables	Cardiovascular disease (n = 149,453)		Respiratory disease (n = 453,868)	
	Daily mean ± SD	Range (daily max–min)	Daily mean ± SD	Range (daily max–min)
**All cases**	133.44 ± 14.60	105	405.24 ± 155.86	1,725
**Age of <65 years**	68.04 ± 9.17	62	368.53 ± 149.29	1,608
**Age of ≥ 65 years**	65.40 ± 9.66	66	36.71 ± 13.52	121
**Male**	74.50 ± 10.11	64	220.18 ± 79.44	816
**Female**	58.94 ± 8.66	60	185.06 ± 78.38	917
**Warm season** **(March–August)**	68.38 ± 15.25	105	205.58 ± 122.71	650
**Cool season** **(September–February)**	65.05 ± 13.86	89	199.65 ± 184.53	1,725

SD: standard deviation.

Table 2 summarizes the air pollution and meteorological data from the study period. The daily average concentrations of PM_10_ and PM_2.5_ were 43.61 μg/m^3^ and 22.01 μg/m^3^, respectively. Among the PM components, NO_3_^-^ had the highest daily concentration value (8.12 μg/m^3^), which was followed by OC (7.24 μg/m^3^) and SO_4_^2-^ (6.20 μg/m^3^). The interquartile ranges (IQR) for PM_10_ and PM_2.5_ were 27.05 μg/m^3^ and 13.90 μg/m^3^, respectively. Unlike the average daily concentrations, the highest IQR value was observed for NO_3_^-^ (5.58 μg/m^3^), which was followed by NH_4_^+^ (5.10 μg/m^3^) and OC (3.90 μg/m^3^). Histograms of the PM components’ concentrations are shown in Fig C in S1 File.

**Table 2:** Air pollution and meteorological data from April 17, 2010 to May 10, 2013.

		Daily mean ± SD	Interquartile range
**Air pollution data**	PM_10_ (μg/m^3^)	43.61 ± 18.94	27.05
	PM_2.5_ (μg/m^3^)	22.01 ± 9.84	13.90
	Organic carbon (μg/m^3^)	7.24 ± 2.98	3.90
	Elemental carbon (μg/m^3^)	1.46 ± 0.62	0.90
	SO_4_^2-^ (μg/m^3^)	6.20 ± 3.41	3.85
	NO_3_^-^ (μg/m^3^)	8.12 ± 5.11	5.58
	NH_4_^+^ (μg/m^3^)	5.84 ± 3.86	5.10
**Meteorological data**	Temperature (°C)	12.17 ± 11.10	19.5
	Relative humidity (%)	59.14 ± 15.38	22.48

SD: standard deviation.

Table 3 shows the Pearson correlation coefficients for the concentrations of PM components. The strongest correlation with PM_2.5_ was observed for SO_4_^2-^ (r = 0.73). Among the PM components, OC and EC were the strongest correlations (r = 0.79). The weakest correlation was between EC and NH_4_^+^ (r = 0.1).

**Table 3:** Pearson correlation coefficients among concentrations of PM components.

	PM_10_	PM_2.5_	Organic carbon	Elemental carbon	SO_4_^2-^	NO_3_^-^	NH_4_^+^
**PM**_**10**_	1.00	0.88	0.49	0.42	0.60	0.64	0.37
**PM**_**2.5**_		1.00	0.50	0.41	0.73	0.69	0.38
**Organic carbon**			1.00	0.79	0.26	0.40	0.12
**Elemental carbon**				1.00	0.28	0.31	0.10
**SO**_**4**_^**2-**^					1.00	0.63	0.58
**NO**_**3**_^**-**^						1.00	0.56
**NH**_**4**_^**+**^							1.00

**Fig 1:**
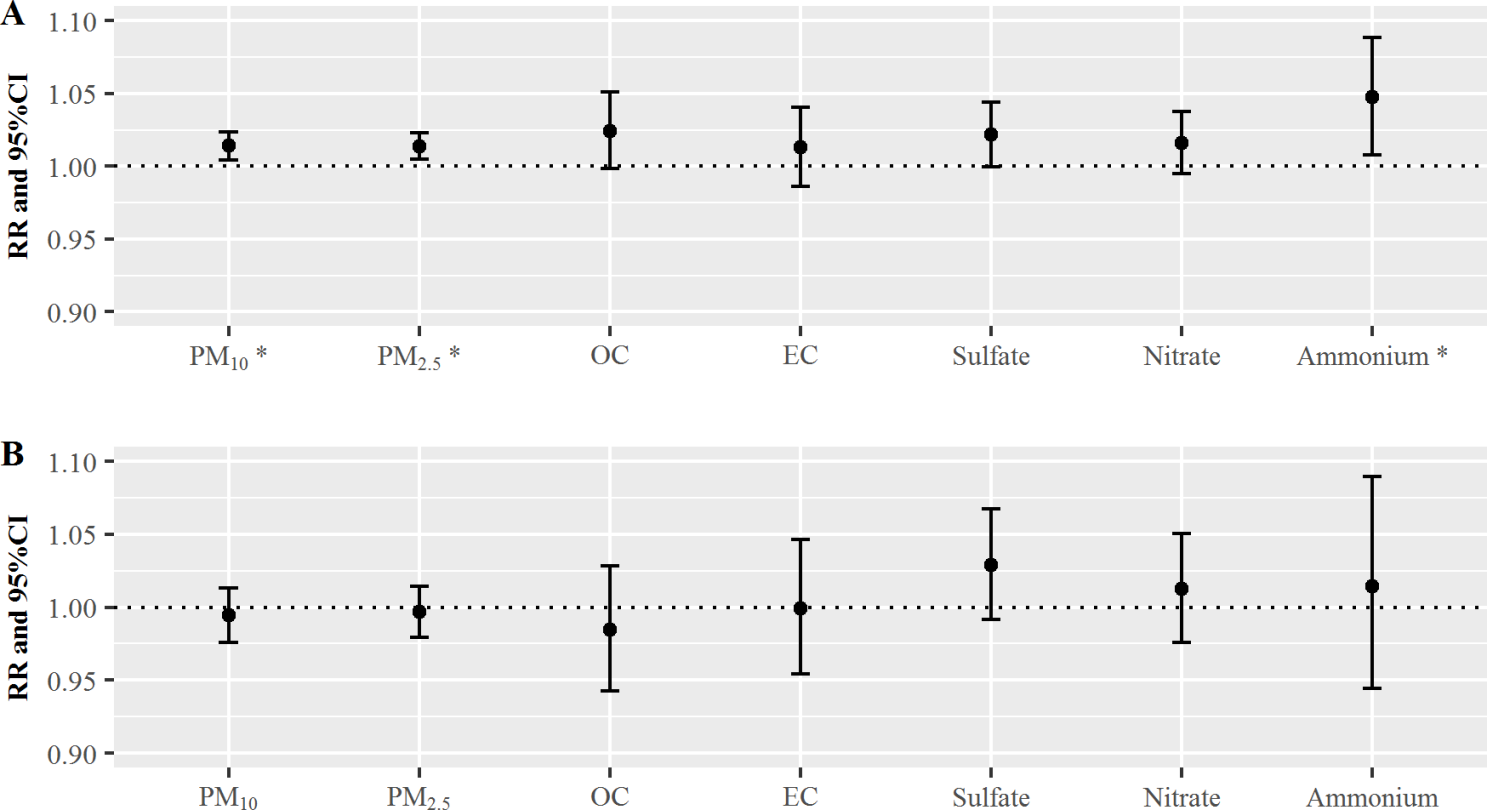
**The relative risks (RRs) of cardiovascular (A) and respiratory (B) diseases on lag 0 day per one-interquartile range increase in PM**_**2.5**_ **and its components.** * PM_10_, PM_2.5_, and ammonium were associated with increased risks of cardiovascular disease. CI: confidence interval, OC: organic carbon, EC: elemental carbon. Lag 0 day indicates that the RRs were measured on the day of exposure.

Fig 1 shows the relative risks (RR) of cardiovascular (Fig 1A) and respiratory (Fig 1B) diseases on lag 0 day when exposed to the PMs and their components. The PM_10_, PM_2.5_, and component variables exhibited positive associations with ER visits for cardiovascular disease. The estimated RRs on lag 0 day for PM_10_ and PM_2.5_ were both 1.01 (95% confidence interval [CI]: 1.00–1.02). The estimated RRs on lag 0 day for OC and EC were 1.02 (95% CI: 1.00–1.05) and 1.01 (95% CI: 0.99–1.04), respectively. The estimated RR on lag 0 day for NH_4_^+^ was 1.05 (95% CI: 1.01–1.09). As shown in Fig 1B, the estimated RRs of respiratory ER visits due to SO_4_^2-^ and NO_3_^-^ in PM_2.5_ were 1.03 (95% CI: 0.99–1.07) and 1.01 (95% CI: 0.98–1.05), respectively. The largest RR estimate was observed for SO_4_^2-^ (Fig 1B), which was followed by NH_4_^+^, NO_3_^-^_,_ and EC. Interestingly, the association of respiratory ER visits with SO_4_^2-^ was stronger than that for cardiovascular disease on lag 0 day. The details regarding the RR values in Fig 1 are summarized in Table A in S1 File. In addition, we analyzed multi-pollutant models for each PM_2.5_ components. For cardiovascular disease, the highest RR on lag 0 day was observed for SO_4_^2-^ (RR: 1.03, 95% CI: 0.98–1.07). For respiratory disease, the highest RR on lag 0 day was observed for EC (RR: 1.09, 95% CI: 0.97–1.23). The estimated RRs of cardiovascular disease in the multi-pollutant models for OC, NO_3_^-^_,_ and NH_4_^+^ were lower than the values from the single-pollutant models. However, only the estimated RR of respiratory disease in the multi-pollutant model for NO_3_^-^ was lower than the value from the single-pollutant model. NH_4_^+^ was not significantly associated with cardiovascular ER visits in the multi-pollutant models, compared to the single-pollutant models. The details regarding the RR values in the multi-pollutant models are summarized in Table B in S1 File.

**Fig 2:**
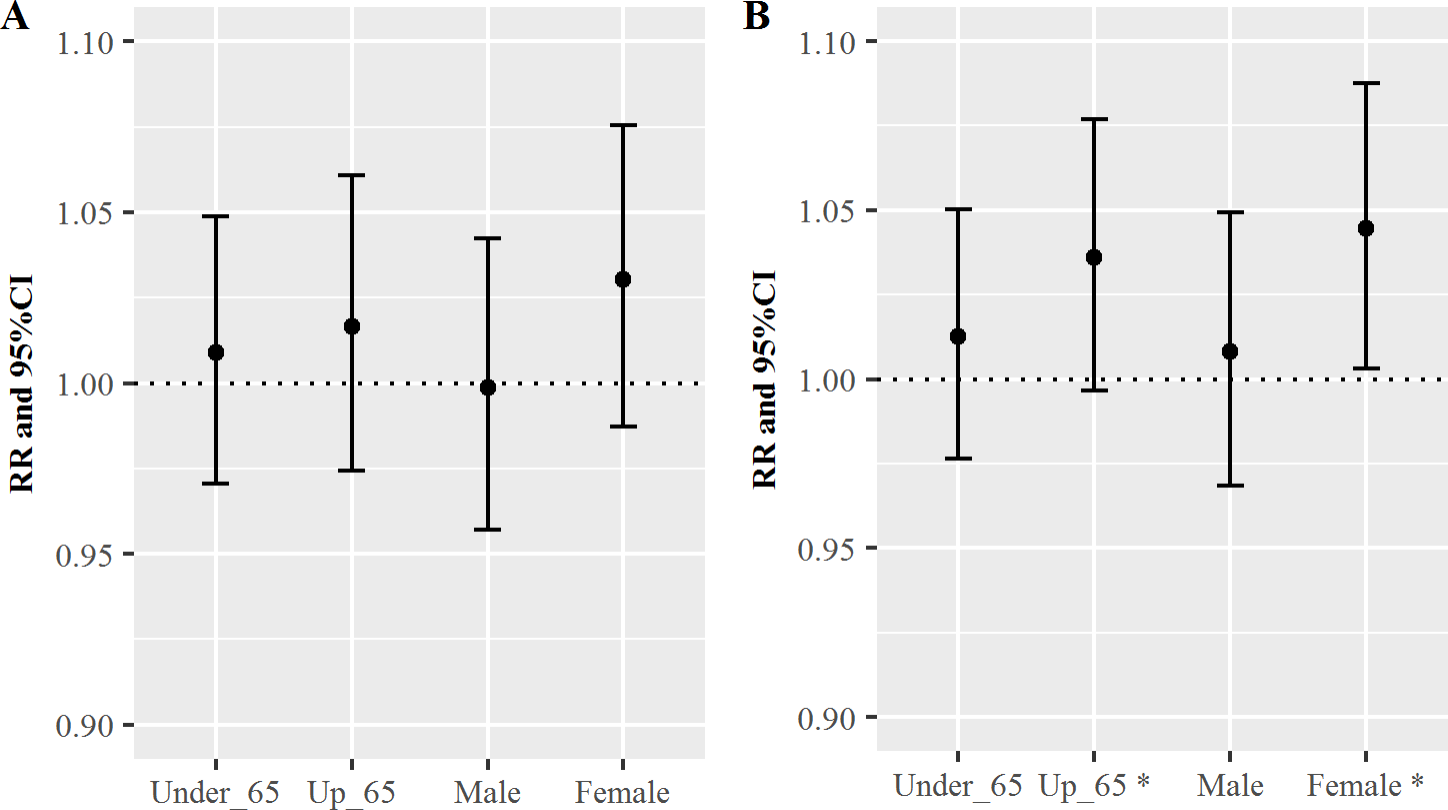
**The relative risks (RRs) of cardiovascular disease per one-interquartile range increase in elemental carbon (EC) (A) or organic carbon (OC) (B) for each age group and sex on lag 0 day.** CI: confidence interval.

Fig 2 shows the RR values for cardiovascular disease per one-IQR increase in OC (Fig 2A) and EC (Fig 2B) for each age group and sex on lag 0 day. Both OC and EC exhibited positive associations with cardiovascular ER visits. The estimated RRs on lag 0 day for OC in the young and elderly groups were 1.01 (95% CI: 0.98–1.05) and 1.04 (95% CI: 1.00–1.08), respectively. The estimated RRs on lag 0 day for OC in the male and female groups were 1.01 (95% CI: 0.97–1.05) and 1.04 (95% CI: 1.00–1.09), respectively (Fig 2A). Similarly, the estimated RRs on lag 0 day for EC in the young and elderly groups were 1.00 (95% CI: 0.97–1.04) and 1.02 (95% CI: 0.98–1.06), respectively. The estimated RRs on lag 0 day for EC in the male and female groups were 1.00 (95% CI: 0.96–1.04) and 1.03 (95% CI: 0.99–1.08), respectively (Fig 2B). The elderly and female groups exhibited greater RR estimates for EC, compared to OC. The RRs for EC were not significant, although the RRs for OC were significant. In addition, we examined the statistical tests for the comparison, and the choice of the test was introduced by Clogg et al. [35] and Paternoster et al. [36]. The differences in the coefficients for elderly and younger patients, and for male and female patients, were not significant for both OC and EC. The information regarding Fig 2 is summarized in Table C in S1 File, and the information regarding the difference testing is summarized in Table D in S1 File.

**Fig 3:**
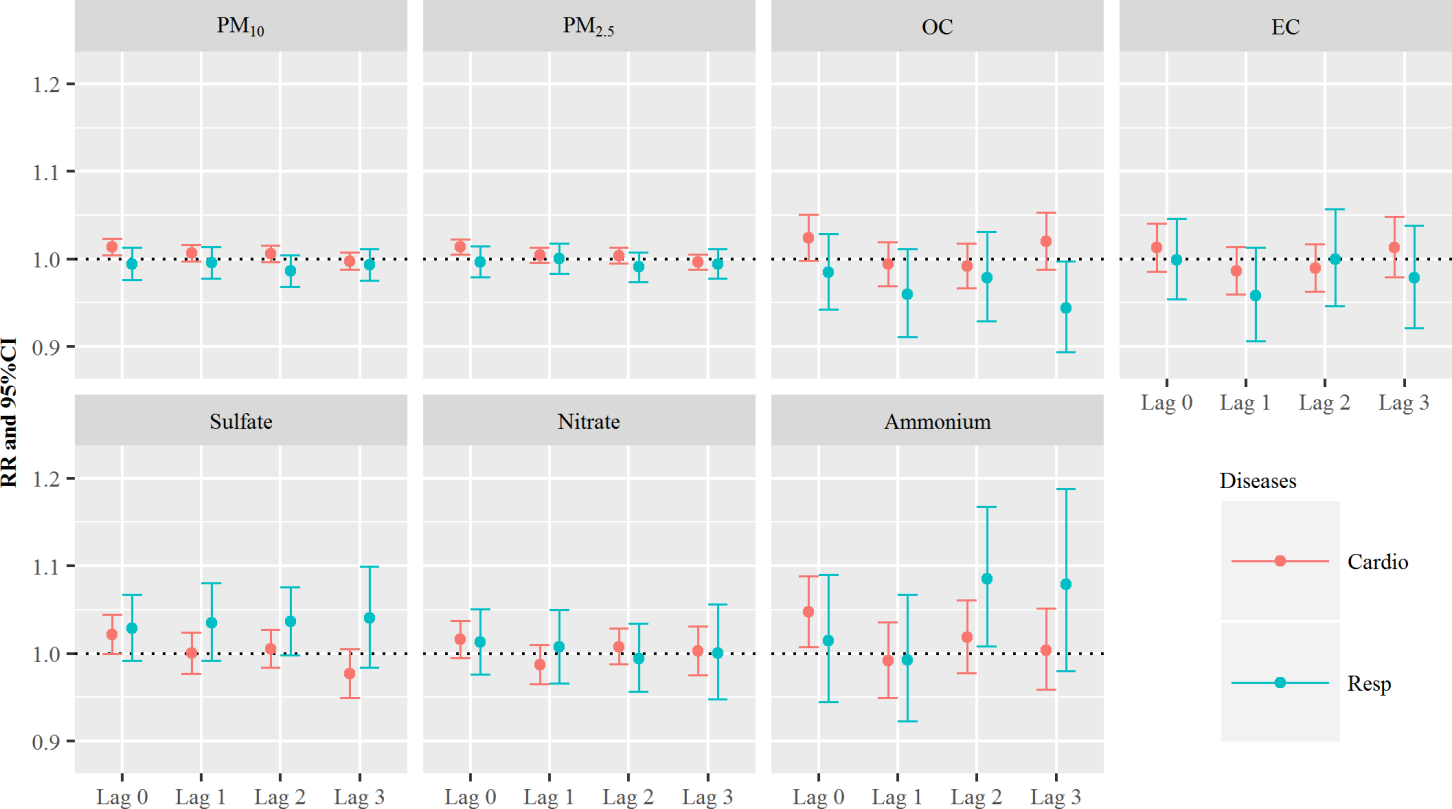
The relative risks (RRs) of cardiovascular and respiratory diseases per one-interquartile range increase in particulate matter and the components on lag 0–3 days.

Fig 3 shows the RR values for cardiovascular and respiratory diseases due to PM and their components, while considering the lag. Based on the results in Fig 1B, exposure to SO_4_^2-^ was positively associated with respiratory ER visits. The estimated RRs per one-IQR increase in SO_4_^2-^ were 1.03 on lag 0 day (95% CI: 0.99–1.07) and 1.04 on lag 1 day (95% CI: 0.99–1.08). The RRs in SO_4_^2-^ slightly increased up to lag 3. We also stratified the analyses according to age and sex, and the results revealed that SO_4_^2-^ had the greatest effect on health outcomes in the elderly group on lag 1 day (RR: 1.05, 95% CI: 1.00–1.10). The RRs per one-IQR increase in SO_4_^2-^ for respiratory ER visits for age groups, sex, and lag days are summarized in Table E in S1 File. All information regarding the stratified analyses are described in Fig D in S1 File.

We evaluated the statistical models by changing the df for time and temperature, and observed a maximum change in the RR of 0.62% (Table F in S1 File).

## 4. Discussion

The present study is the first to investigate the relationship between cardiovascular/respiratory diseases and PM components in Seoul, South Korea. Our findings indicate that PMs and their components were more positively associated with ER visits for cardiovascular disease, compared to those for respiratory disease, and these findings were consistent with findings from previous studies [21, 28, 37]. Peng et al. [21] reported that nitrate, EC, OC, and ammonium could increase the risk of ER visits for cardiovascular disease, with ammonium exhibiting the strongest association. Peng et al. [21] estimated that one-IQR increases in EC, OC, and ammonium were associated with 0.72% (95% CI: 0.43–1.01%), 0.66% (95% CI: 0.29–1.02%), and 0.68% (95% CI: 0.31–1.06%) increases in cardiovascular hospital admissions. Another study investigated the associations of ER visits for specific cardiovascular diseases with PM components, and revealed that exposure to EC and OC may induce ER visits [28]. For example, EC exposure increased the risk of ER visits for ischemic and hemorrhagic stroke (RR: 0.99 per 1.0 μg/m^3^, 95% CI: 0.95–1.03 and RR: 1.07 per 1.0 μg/m^3^, 95% CI: 1.00–1.14; respectively). Similar results were observed for OC exposure (RR: 1.03 per 3.0 μg/m^3^, 95% CI: 0.98–1.09 and RR: 1.10 per 3.0 μg/m^3^, 95% CI: 0.99–1.22, respectively). Kim et al. [35] also reported that sulfate, EC, and OC were associated with increasing risks of cardiovascular ER visits. Those authors adjusted the lag models for 14 days and estimated the cumulative RRs for cardiovascular hospital admissions on lag 0–1 day, and reported that one-IQR increases in sulfate, EC, and OC provided RRs of 1.004 (95% CI: 0.995–1.013), 1.018 (95% CI: 1.006–1.030), and 1.015 (95% CI: 1.002–1.029), respectively. However, a recent study by Wang and Lin [38] revealed that the lag effects of sulfate varied according to the cause-specific ER visits. For respiratory disease, the estimated RRs decreased until lag 2 day and then increased. Although we observed a different trend (slightly increasing RRs until lag 3 day), both their study and our study revealed positive associations after considering lag effects.

The present study also revealed that EC and OC were associated with more frequent ER visits for both cardiovascular and respiratory diseases among elderly and female patients. Previous studies regarding the biological mechanisms for these relationships support our finding [39–43], and inflammation that is induced by EC/OC may be responsible for the cardiovascular ER visits. In this context, EC exposure may decrease the myocardial oxygen supply and increase the risk of cardiac ischemia [42, 43], which may induce cardiac disease through potentially interrelated mechanisms, including systemic inflammation and oxidative stress [42, 43]. Moreover, EC exposure is associated with ST-segment depression, which possibly represents myocardial ischemia or inflammation [41]. Delfino et al. [39] have reported that OC may be associated with higher concentrations of nitric oxide in exhaled breath, which might disrupt cardiovascular function. Seaton et al. [44, 45] have also proposed that fine particles (e.g., OC) could provoke pulmonary inflammation by inducing oxidative stress, which could lead to increased blood coagulability and systemic inflammation [44, 45].

The present study has several limitations. First, the sampling of PM_2.5_ and the PM components was performed over a period of several days, because of the sampling schedule at the Atmospheric Environment & Climate Change laboratory in Seoul National University. This prevented us from using a distributed lag model, because the data were not measured every day. However, we applied the moving averages concept to consider the short-term lag effects, which allows us to investigate the short-term associations between PM components and ER visits. Another limitation is that we used stationary air pollution data, rather than the individual’s exposures to PM and PM components, as it is difficult to examine the influence of air pollution exposure at the individual level. This approach may have introduced random errors in the PM component data and led to a reduction in the related RR values [46]. In addition, PM components influence people when they are near ground level, while we collected PM component data on a rooftop of a large building, which may have created discordance between the air pollutant data and the exposure location. Furthermore, we could not consider repeated admissions for the same person because our ER visit data were anonymized, and our results may have been overestimated because we could not address the correlated data [47].

Our findings regarding the influences of EC, OC, and sulfate suggest that traffic- and industrial-related sources play important roles in driving ER visits for cardiovascular and respiratory diseases. In this context, sulfate is emitted from sulfur in diesel fuel [48]. Similarly, the major sources of EC and OC are related to urban development, such as motorized vehicles, coal burning, shipping emissions, and industrial sources [49]. As Seoul is a representative traffic-congested region in South Korea, studies regarding the health effects of traffic-related air pollution have recently increased in importance. Thus, our data may help promote further research regarding the effects of PM components, and may facilitate the control and regulation of air pollution.

## 5. Conclusions

Our analyses revealed that PM_2.5_ mass and PM_2.5_ components were associated with cardiovascular ER visits, with the strongest association observed for NH_4_^+^. When we performed stratified analyses according to age and sex, OC was significantly associated with cardiovascular ER visits among elderly and female patients. Similarly, EC was positively associated with cardiovascular ER visits among elderly and female patients. Lagged SO_4_^2-^ was associated with respiratory ER visits. Based on our results, it appears that elderly and female patients are more vulnerable to both OC and EC. Furthermore, as SO_4_^2-^, EC, and OC are generally categorized as traffic-related air pollutants, our findings may facilitate health policy development and promote the management of traffic-related air pollutants.

## Supporting information

S1 File Supplemental tables and figures. (ZIP)
