# Supplementary material for: Associations of particulate matter and its components with emergency room visits for cardiovascular and respiratory diseases
Source: PLoS One. 2017 Aug 15;12(8):e0183224. doi: 10.1371/journal.pone.0183224 (PMC5557583; doi:10.1371/journal.pone.0183224)
Supplement: S1 File — (ZIP) [file pone.0183224.s001.zip › S1 File_supplementary tables and figures_sunghee_journal_20170331.docx]

**Table A. Relative risks per one IQR increase of air pollutants on cardiovascular disease and respiratory ER visits for the concurrent day.**

| Air pollution | IQR  (𝜇g/m^3^) | Cardiovascular diseases | | | Respiratory diseases | | |
| --- | --- | --- | --- | --- | --- | --- | --- |
|  |  | RR | 95% CI | P-value | RR | 95% CI | P-value |
| PM_10_ | 27.05 | 1.01* | (1.00,1.02) | 0.004 | 0.99 | (0.98,1.01) | 0.573 |
| PM_2.5_ | 13.90 | 1.01* | (1.00,1.02) | 0.002 | 1.00 | (0.98,1.01) | 0.711 |
| OC | 3.90 | 1.02 | (1.00,1.05) | 0.069 | 0.98 | (0.94,1.03) | 0.491 |
| EC | 0.89 | 1.01 | (0.99,1.04) | 0.036 | 1.00 | (0.95,1.05) | 0.975 |
| SO_4_^2^ | 3.85 | 1.02 | (1.00,1.04) | 0.057 | 1.03 | (0.99,1.07) | 0.133 |
| NO_3_^-^ | 5.58 | 1.02 | (1.00,1.04) | 0.144 | 1.01 | (0.98,1.05) | 0.503 |
| NH_4_^+^ | 5.09 | 1.05* | (1.01,1.09) | 0.023 | 1.01 | (0.94,1.09) | 0.693 |

CI, confidence interval; IQR, interquartile range; RR, relative risk

* p-value of RRs < 0.05

**Table B. Relative risks (RRs) per one IQR increase of each PM_2.5_ components on cardiovascular disease and respiratory ER visits in multi pollutant models.**

| Variables | Air pollution | RR | 95% CI | P-value |
| --- | --- | --- | --- | --- |
| Cardiovascular diseases | **OC** | 1.01 | (0.93 , 1.09) | 0.84 |
|  | **EC** | 1.02 | (0.95 , 1.10) | 0.56 |
|  | **SO_4_^2-^** | 1.03 | (0.98 , 1.07) | 0.28 |
|  | **NO_3_^-^** | 0.99 | (0.95 , 1.04) | 0.80 |
|  | **NH_4_^+^** | 1.00 | (0.94 , 1.07) | 0.93 |
| Respiratory diseases | **OC** | 0.98 | (0.86 , 1.12) | 0.79 |
|  | **EC** | 1.09 | (0.97 , 1.23) | 0.16 |
|  | **SO_4_^2-^** | 0.99 | (0.92 , 1.07) | 0.82 |
|  | **NO_3_^-^** | 1.00 | (0.93 , 1.07) | 1.00 |
|  | **NH_4_^+^** | 0.92 | (0.82 , 1.03) | 0.17 |

**Table C. Relative risks per one IQR increase of OC and EC on cardiovascular ER visits by age and gender**.

| Air pollution | Variables | Cardiovascular diseases | | | Respiratory diseases | | |
| --- | --- | --- | --- | --- | --- | --- | --- |
|  |  | RR | 95% CI | P-value | RR | 95% CI | P-value |
| OC | Age < 65 years old | 1.01 | (0.98,1.05) | 0.50 | 0.99 | (0.94,1.03) | 0.49 |
|  | Age ≥ 65 years old | 1.04** | (1.00,1.08) | 0.08 | 0.98 | (0.93,1.04) | 0.54 |
|  | Male | 1.01 | (0.97,1.05) | 0.69 | 0.99 | (0.94,1.03) | 0.48 |
|  | Female | 1.04* | (1.00,1.09) | 0.04 | 0.98 | (0.94,1.03) | 0.61 |
| EC | Age < 65 years old | 1.01 | (0.97,1.05) | 0.65 | 1.00 | (0.95,1.05) | 0.97 |
|  | Age ≥ 65 years old | 1.02 | (0.97,1.06) | 0.45 | 0.99 | (0.93,1.05) | 0.93 |
|  | Male | 1.00 | (0.96,1.04) | 0.95 | 1.01 | (0.96,1.06) | 0.63 |
|  | Female | 1.03 | (0.99,1.08) | 0.17 | 0.99 | (0.94,1.04) | 0.68 |

* p-value of RRs < 0.05 ** p-value of RRs < 0.10

**Table D. Statistical tests for the comparison by age and gender.**

$$\mathbf{Z=}\frac{\boldsymbol{\beta}_{\mathbf{1}}\mathbf{-}\boldsymbol{\beta}_{\mathbf{2}}}{\sqrt{{\mathbf{(}\mathbf{SE}_{\boldsymbol{\beta}_{\mathbf{1}}}\mathbf{)}}^{\mathbf{2}}\mathbf{+}{\mathbf{(}\mathbf{SE}_{\boldsymbol{\beta}_{\mathbf{2}}}\mathbf{)}}^{\mathbf{2}}}}$$

where $\beta_{1}$ is the coefficient about elderly patients or female, $\beta_{2}$ is the coefficient about younger patients or male, $\mathrm{SE}_{\beta_{1}}$ is the standard error of $\beta_{1}$, and $\mathrm{SE}_{\beta_{2}}$ is the standard error of $\beta_{2}$.

| Air pollution | Variables | Cardiovascular diseases | | | |
| --- | --- | --- | --- | --- | --- |
|  |  | Coefficient | SE | Z-statistics | P-value |
| OC | Age < 65 years old | 0.003 | 0.005 |  |  |
|  | Age ≥ 65 years old | 0.009 | 0.005 | 0.839 | 0.200 |
|  | Male | 0.002 | 0.005 |  |  |
|  | Female | 0.011 | 0.005 | 1.222 | 0.111 |
| EC | Age < 65 years old | 0.010 | 0.022 |  |  |
|  | Age ≥ 65 years old | 0.019 | 0.024 | 0.260 | 0.397 |
|  | Male | -0.001 | 0.024 |  |  |
|  | Female | 0.034 | 0.024 | 1.015 | 0.156 |

SE, standard error

**Table E. Relative risks per one IQR increase of SO_4_^2-^ on respiratory ER visits by age and gender for different lag days**.

| Air pollution | Variables | Lag 0 day | Lag 1 day | Lag 2 day | Lag 3 day |
| --- | --- | --- | --- | --- | --- |
| SO_4_^2-^ | All observations | 1.03  (0.99-1.07) | 1.04  (0.99-1.08) | 1.04  (1.00-1.08) | 1.04  (0.98-1.10) |
|  | Age < 65 years old | 1.03  (0.99-1.07) | 1.03  (0.99-1.08) | 1.04  (1.00--1.08) | 1.04  (0.98-1.11) |
|  | Age ≥ 65 years old | 1.02  (0.98-1.07) | 1.05  (1.00-1.10) | 1.00  (0.96-1.05) | 1.02  (0.96-1.08) |
|  | Male | 1.03  (0.99-1.07) | 1.03  (0.98-1.08) | 1.04  (1.00-1.08) | 1.03  (0.98-1.09) |
|  | Female | 1.03  (0.99-1.07) | 1.04  (1.00-1.09) | 1.04  (0.99-1.08) | 1.05  (0.99-1.11) |

**Table F. Sensitivity of the relative risks (RRs) per one IQR increase of PM_2.5_ and PM_10_ on cardiovascular disease and respiratory ER visits for the concurrent day by changing degree of freedom on temperature/time.**

| Cardiovascular disease | | | | | | |
| --- | --- | --- | --- | --- | --- | --- |
| Modeling Choices |  | df for time | RRs of PM_10_ | % Change | RRs of PM_2.5_ | % Change |
| Base model | With temperature | 6/year | 1.0141 | - | 1.0139 | - |
|  |  | 5/year | 1.0138 | -0.02 | 1.0138 | -0.01 |
|  |  | 7/year | 1.0142 | +0.01 | 1.0140 | +0.01 |
|  |  | 8/year | 1.0141 | +0.01 | 1.0142 | +0.02 |
| Base model | With time | 7/year | 1.0141 | - | 1.0139 | - |
|  |  | 6/year | 1.0146 | +0.05 | 1.0149 | +0.10 |
|  |  | 8/year | 1.0141 | +0.00 | 1.0157 | +0.18 |
|  |  | 10/year | 1.0135 | -0.05 | 1.0152 | +0.13 |

| Respiratory disease | | | | | | |
| --- | --- | --- | --- | --- | --- | --- |
| Modeling Choices |  | df for time | RRs of PM_10_ | % Change | RRs of PM_2.5_ | % Change |
| Base model | With temperature | 6/year | 0.9946 | - | 0.9967 | - |
|  |  | 5/year | 0.9935 | -0.11 | 0.9961 | -0.06 |
|  |  | 7/year | 0.9953 | +0.07 | 0.9971 | +0.04 |
|  |  | 8/year | 0.9952 | +0.06 | 0.9972 | +0.05 |
| Base model | With time | 7/year | 0.9946 | - | 0.9967 | - |
|  |  | 6/year | 0.9934 | -0.13 | 1.0028 | +0.62 |
|  |  | 8/year | 0.9903 | -0.44 | 0.9993 | +0.26 |
|  |  | 10/year | 0.9886 | -0.61 | 0.9923 | -0.44 |


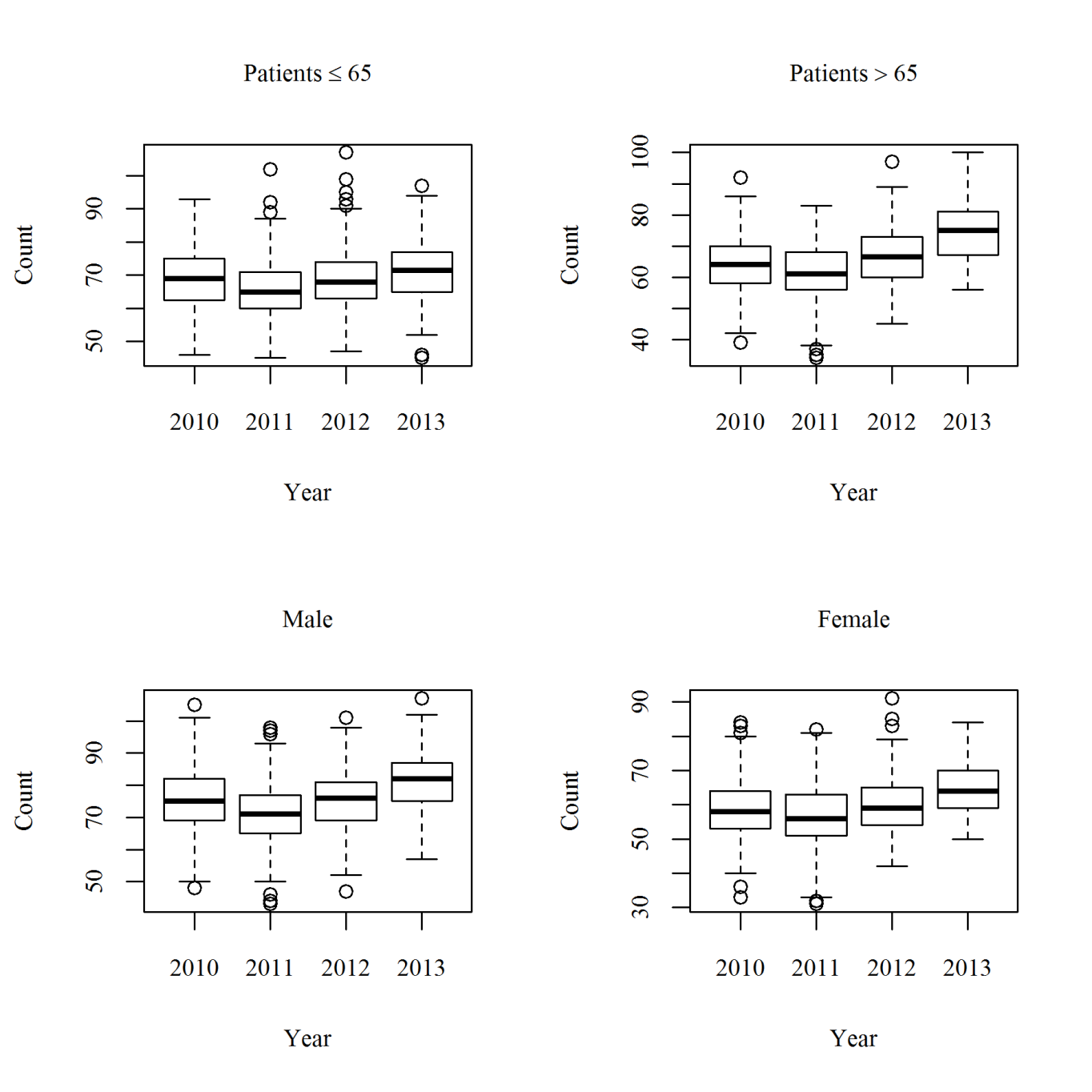
**Fig A. Box plots of ER visits for cardiovascular diseases by age and gender.**

**
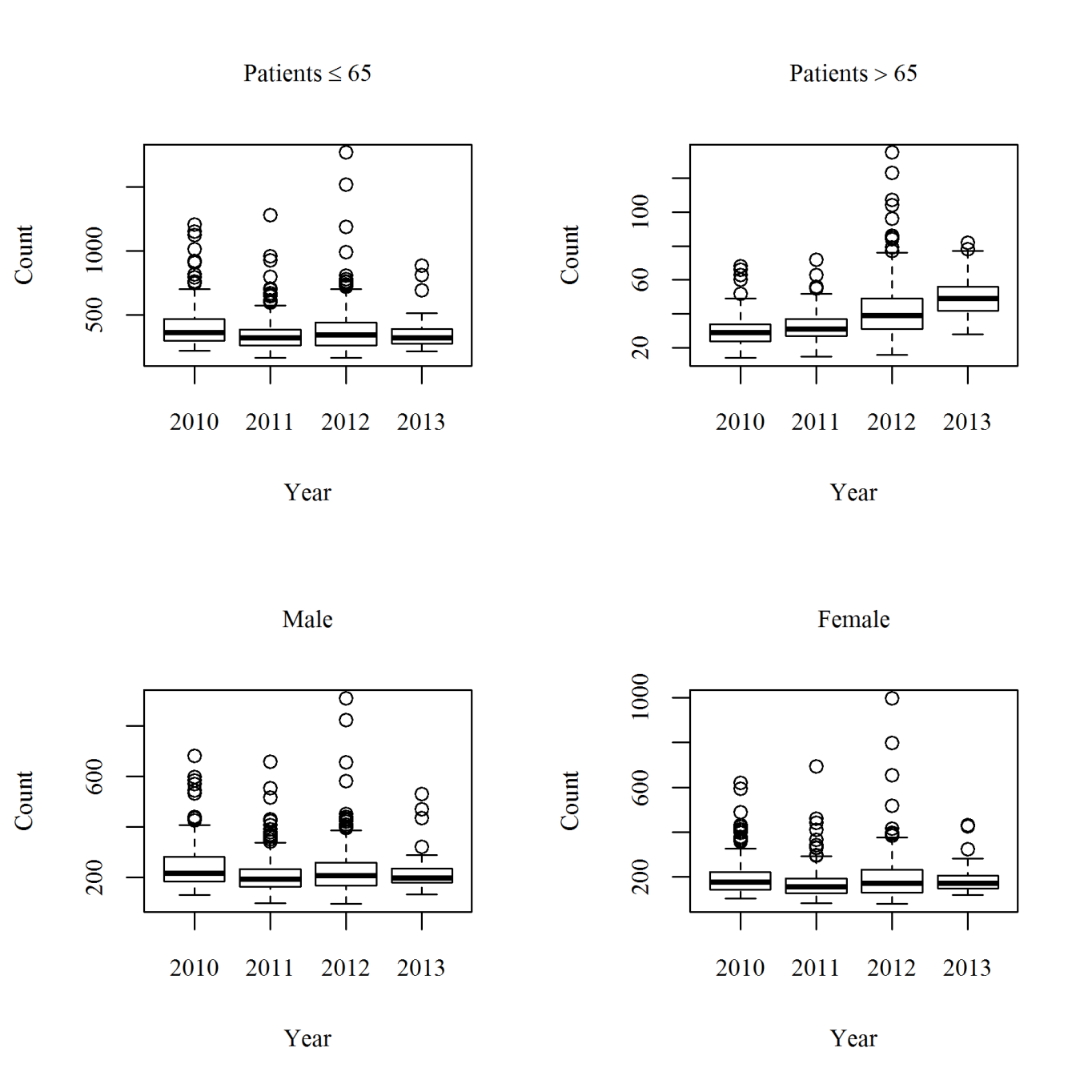
Fig B. Box plots of ER visits for respiratory diseases by age and gender.**


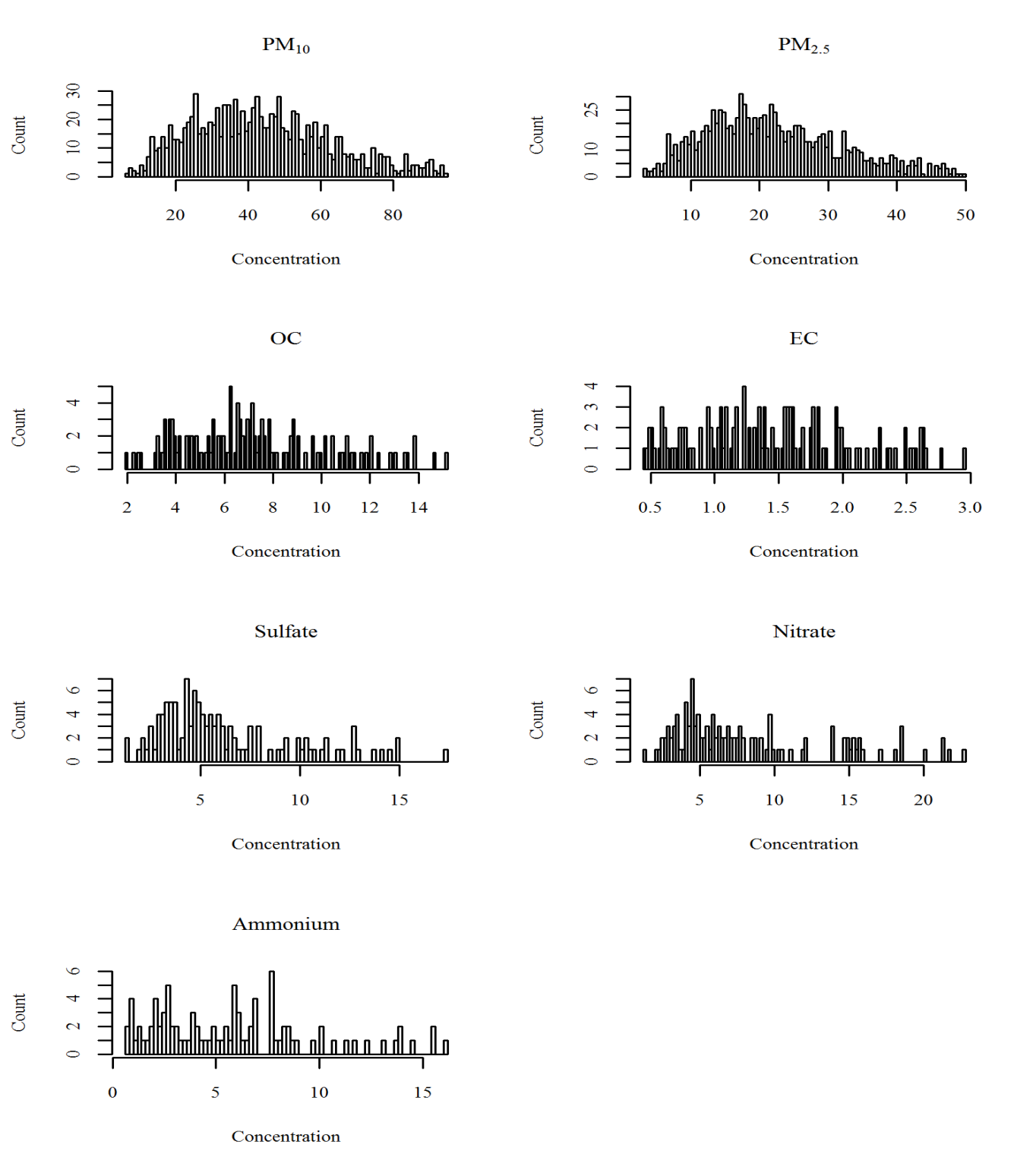


**Fig C. Histograms of PM_2.5_ components’ concentrations**

**
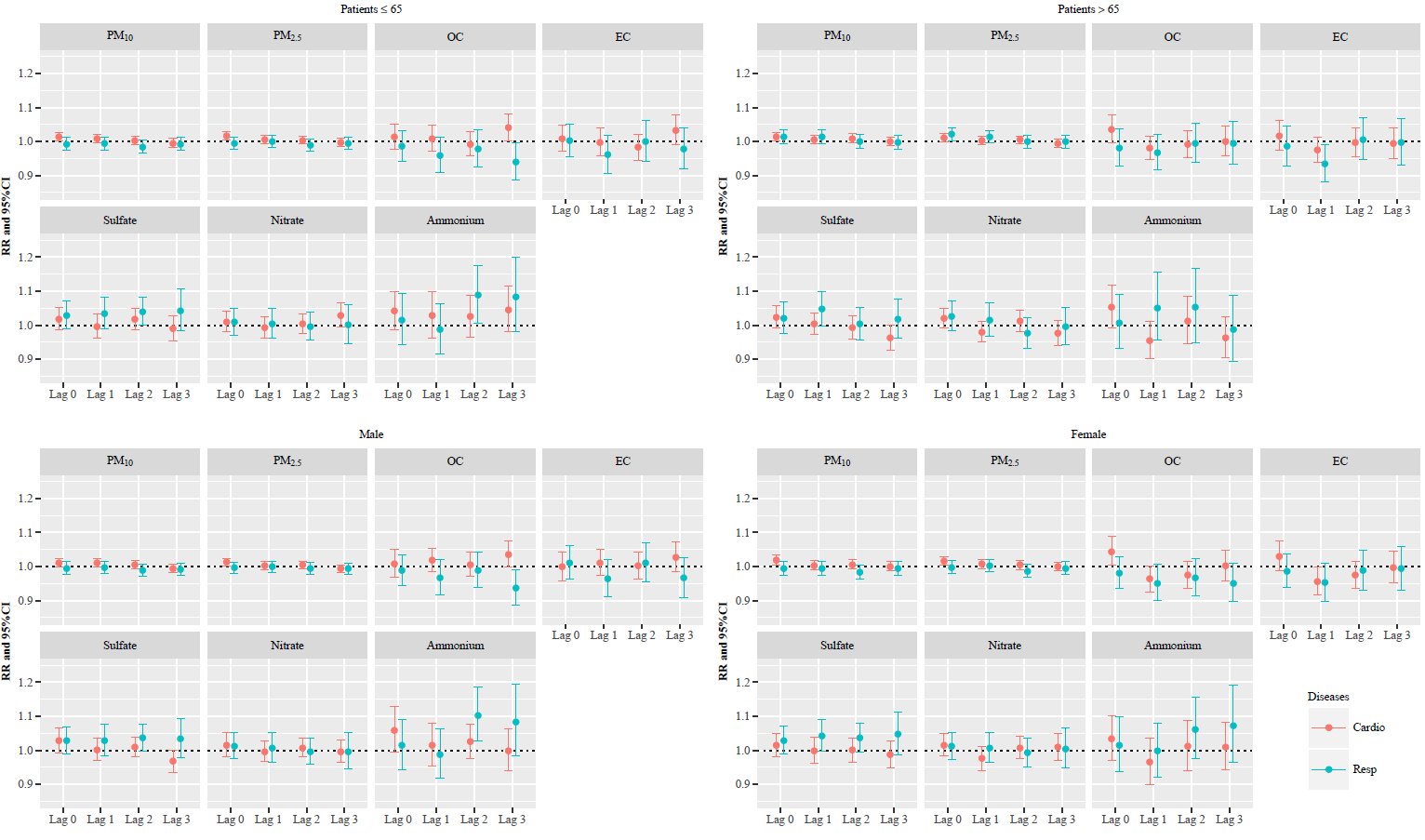
**

**Fig D. RRs per one IQR increase of PM_2.5_ and its components on cardiovascular and respiratory ER visits for different lag days by age and gender.**
