# Supplementary figures and images for: Associations of particulate matter and its components with emergency room visits for cardiovascular and respiratory diseases
Source: PLoS One. 2017 Aug 15;12(8):e0183224. doi: 10.1371/journal.pone.0183224 (PMC5557583; doi:10.1371/journal.pone.0183224)

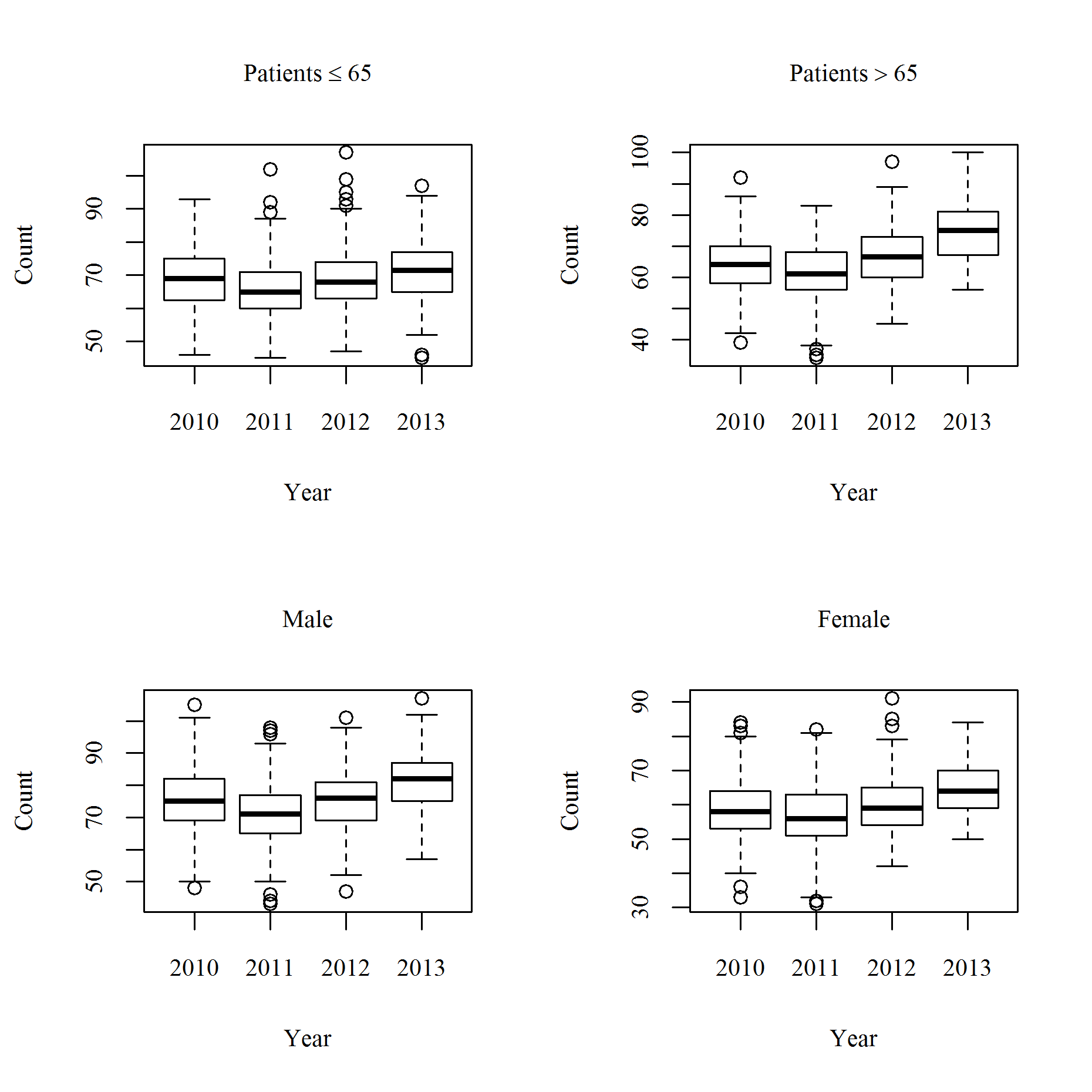

Supplement: S1 File — (ZIP) [file pone.0183224.s001.zip › Fig. A.tif]

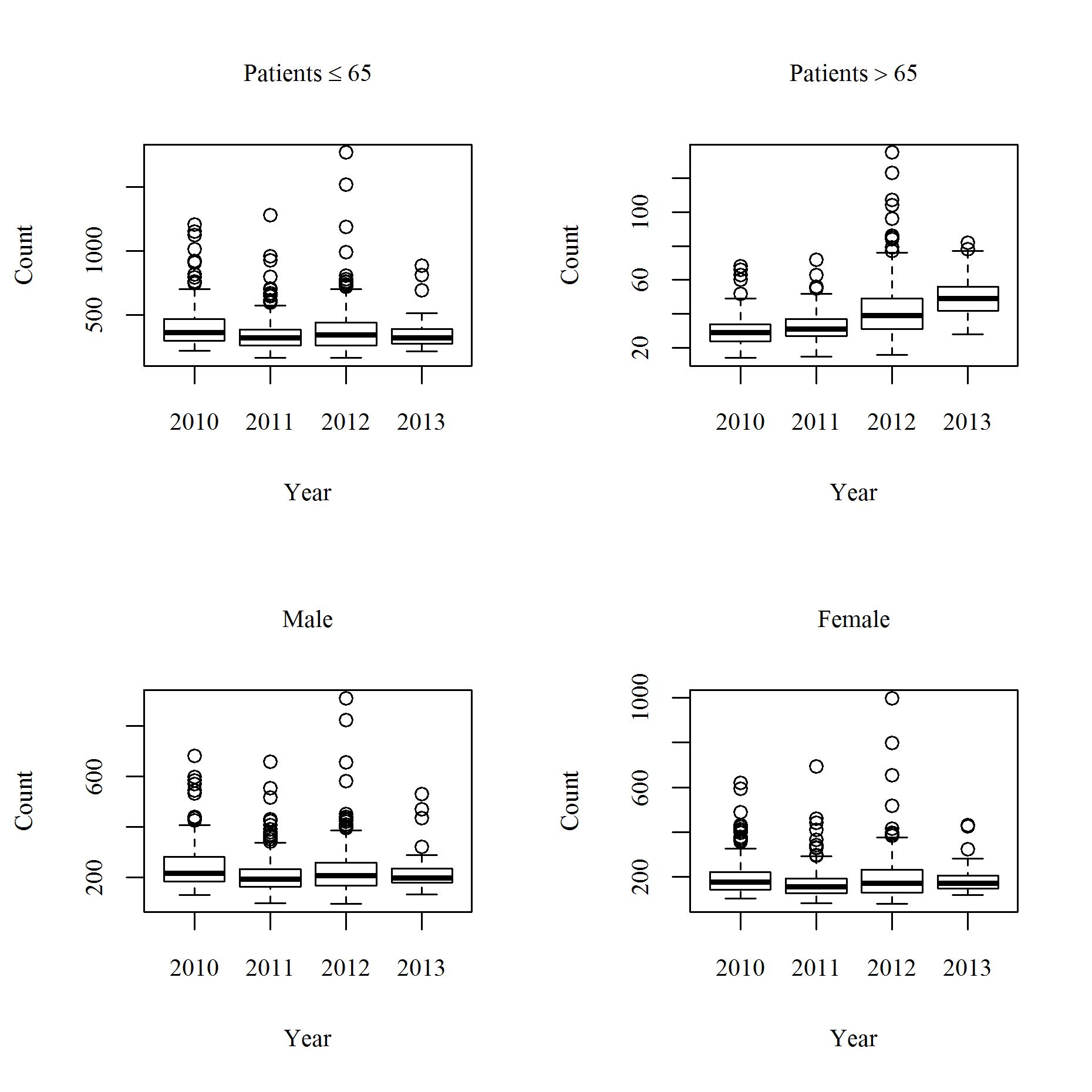

Supplement: S1 File — (ZIP) [file pone.0183224.s001.zip › Fig. B.tif]

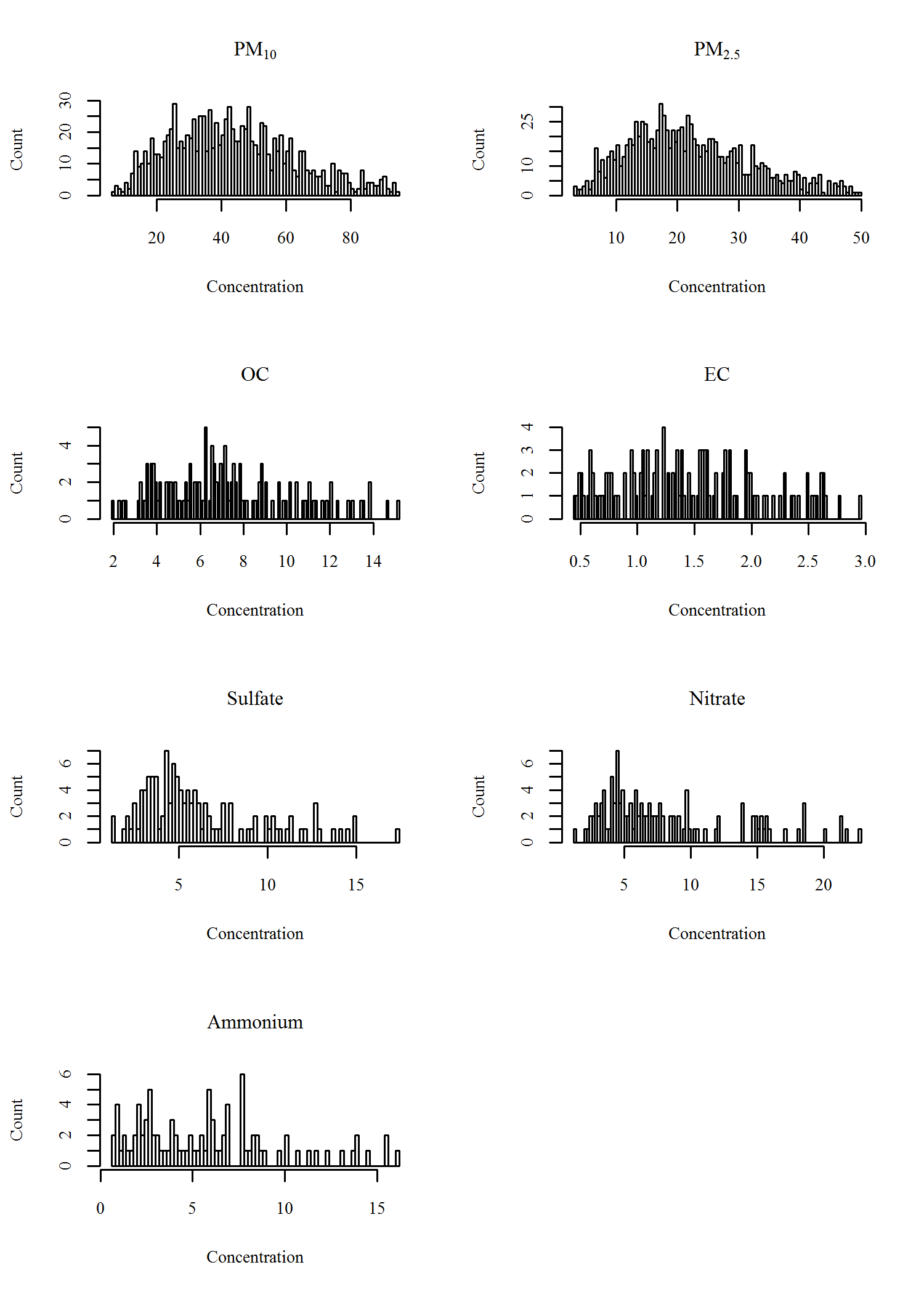

Supplement: S1 File — (ZIP) [file pone.0183224.s001.zip › Fig. C.tif]

Patients  $\leq 65$ 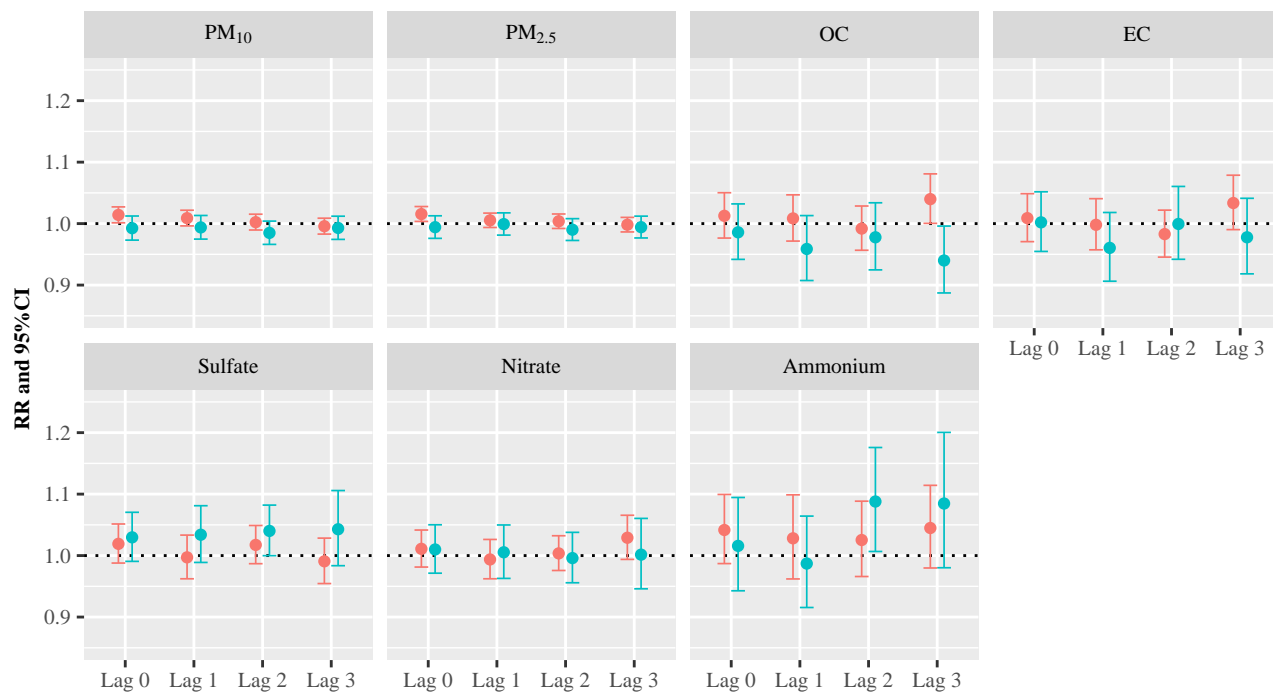Patients  $> 65$ 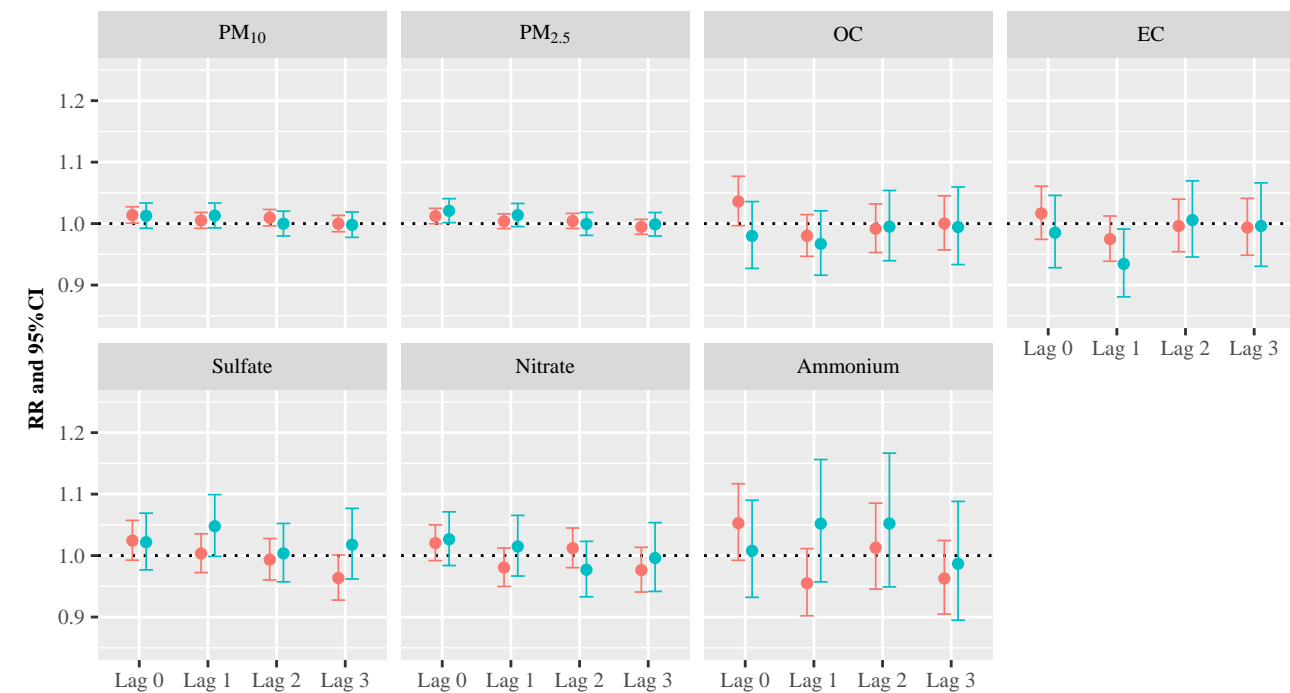

Male

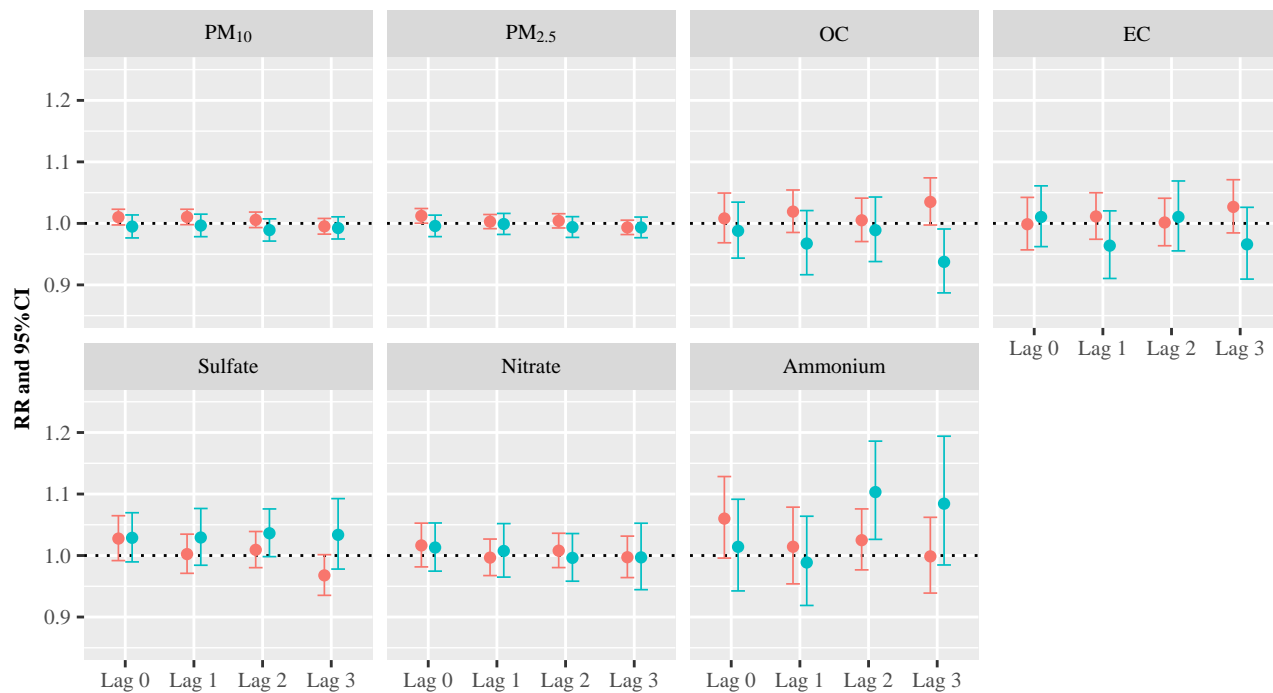

Female

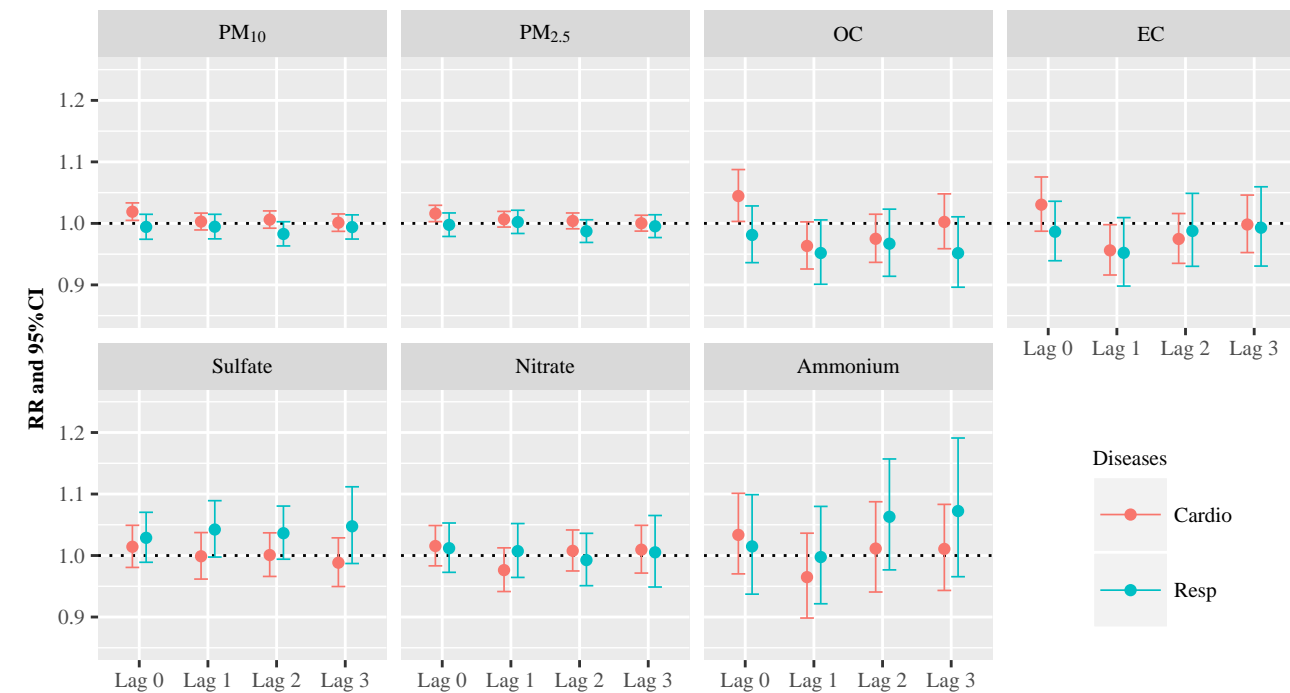

Diseases

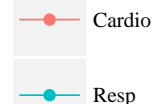

Supplement: S1 File — (ZIP) [file pone.0183224.s001.zip › Fig. D.pdf]
